# Procedures for risk management and a review of crisis referrals from the MindSpot Clinic, a national service for the remote assessment and treatment of anxiety and depression

**DOI:** 10.1186/s12888-015-0676-6

**Published:** 2015-12-01

**Authors:** Olav Nielssen, Blake F. Dear, Lauren G. Staples, Rebecca Dear, Kathryn Ryan, Carol Purtell, Nickolai Titov

**Affiliations:** MindSpot Clinic, 16 University Ave, North Ryde, NSW 2019 Australia; University of Sydney, 92 Parramatta Road, Camperdown, NSW 2050 Australia; University of New South Wales, Anzac Pde Kensington, NSW 2033 Australia; Macquarie University, Balaclava Road, North Ryde, NSW 2019 Australia

**Keywords:** MindSpot, Anxiety, Depression, Risk assessment, Suicide, Online treatment, Crisis

## Abstract

**Background:**

The MindSpot Clinic (MindSpot) provides remote screening assessments and therapist-guided treatment for anxiety and depression to adult Australians. Most patients are self-referred. The purpose of this study was to report on the procedures followed to maintain the safety of patients and to examine the circumstances of urgent referrals to local services made by this remote mental health service.

**Method:**

A description of the procedures used to manage risk, and an audit of case summaries of patients who were urgently referred for crisis intervention. The reported measures were scores on self-report scales of psychological distress (K-10) and depression (PHQ-9), the number reporting suicidal thoughts and plans, and the number of acute referrals.

**Results:**

A total of 9061 people completed assessments and consented for analysis of their data in the year from 1 July, 2013 to 30 June, 2014. Of these, 2599 enrolled in online treatment at MindSpot, and the remainder were supported to access local mental health services. Suicidal thoughts were reported by 2366 (26.1 %) and suicidal plans were reported by 213 (2.4 %). There were 51 acute referrals, of whom 19 (37.3 %) lived in regional or remote locations. The main reason for referral was the patients’ self-report of imminent suicidal intent. The police were notified in three cases, and in another case an ambulance attended after the patient reported taking an overdose. For the remaining acute referrals, MindSpot therapists were able to identify a local mental health service or a general practitioner, confirm receipt of a written case summary, and confirm that the patient had been contacted, or that the local service intended to contact the patient.

**Conclusions:**

Around 0.6 % of the people seeking assessment or treatment by MindSpot were referred to local mental health services for urgent face to face care. The procedures for identifying and managing those patients were satisfactory, and in every case, either emergency services or local mental health services were able to take over the patient’s care. This review suggests that the uncertainty associated with taking responsibility for the remote treatment of patients who disclose active suicidal plans is not a major impediment to providing direct access online treatment for severe forms of anxiety and depression.

**Electronic supplementary material:**

The online version of this article (doi:10.1186/s12888-015-0676-6) contains supplementary material, which is available to authorized users.

## Background

Community surveys have consistently identified that less than half of all people with clinically significant levels of anxiety and depression receive any form of treatment [1, 2], and only a portion of those receive evidence based care [3]. There is also a growing body of evidence showing the effectiveness of cognitive behaviour therapy delivered via the internet [4, 5]. The MindSpot Clinic (MindSpot) was funded by the Australian Federal Government as part of the eMental Health Strategy for Australia [6] of which one key aim was to increase access to evidence-based mental health services for Australian adults with anxiety and depression.

Supporting patient safety and identifying patients who might go on to commit suicide is a key challenge and major concern of all mental health services. An important task of MindSpot is to identify patients who are at immediate risk of suicide and to refer them for appropriate treatment or support. However, MindSpot differs from many other remote or online treatment services because most referrals come directly from the public, rather than from a referring agency that knows the patient and can intervene and arrange emergency care for suicidal behaviour if required. Moreover, MindSpot assesses and treats people from all over Australia from facilities on the campus of Macquarie University in Sydney, between the hours of 8:00 am to 8:00 pm from Monday to Saturday and relies on local mental health services from all over Australia to provide emergency care. A major priority in the service planning and the development of operational policies and procedures has been the safety of patients, how best to identify patients at risk of suicide and how the service should respond to those patients.

The risk management procedures at MindSpot have been developed in accordance with a best practice model, drawing from guidelines developed by the New South Wales Ministry of Health (http://www.health.nsw.gov.au/mhdao/publications/Pages/suicide-risk.aspx), and rely on the self report of suicidal thoughts and plans to identify people who are contemplating suicide. However, a recent meta-analysis found that expressed suicidal ideation is not associated with subsequent suicide in patients with a diagnosis of mood disorder [7], and several other studies have shown limits in the ability of risk assessment procedures to predict which patients will commit suicide. For example, studies of factors associated with the suicide of inpatients [8] and patients discharged from psychiatric hospitals [9] have shown that the base rate of suicide is too low, even in these high risk groups, and the factors associated with suicide are too common, to be able to use risk algorithms to identify which patients might go on to commit suicide in order to intervene to prevent suicide. The alternative to attempting to predict which patients will commit suicide is to provide an adequate standard of care to all patients, including supporting all patients to develop a pragmatic safety plan, referral to local services for those in acute distress and providing effective treatment for depression to people who would not otherwise receive any treatment.

Other remote services have considered the issue of how best to manage patients at risk and the liability for adverse events among patients treated remotely. For example, Fitzgerald et al. [10] noted the requirement to provide competent care in a way that did not harm the patient, and also the duty to try to protect patients in crisis. They recommended the development of policies to manage crises, and recording all contact and treatment plans. In a trial of a web based self help intervention for suicidal thoughts, van Spijker et al. reviewed the safety of patients using twice weekly enquiries regarding the presence of suicidal thoughts and intervention in response to elevated scores on scales for assessing suicidal ideation and depression [11]. A recent consensus statement on defining and measuring negative effects of internet interventions by pioneering treatment providers in Sweden, the Netherlands, Germany and Australia [12] noted the lack of research on negative effects of internet provided treatment when compared to those of face to face psychotherapy. The paper raised a number of hypothetical reasons for negative effects, including disappointment, deficient preparation and treatment, and patient and therapist characteristics. The statement recommended regular measurement of symptoms and outcome, and intervention in patients who deteriorate or have high symptom scores and fail to progress in treatment.

Consistent with this literature, MindSpot has developed procedures to actively manage the risk of self-harm by developing Safety Plans for all patients, the use of weekly automated assessments of symptoms and patient safety, and a framework to ensure consistent responses to patients whose communication with the service indicates that they might be at risk.

The aim of this paper was to describe the safety procedures of MindSpot and to review the characteristics and circumstances of patients referred for urgent face to face mental health assessment.

## Methods

We report on the procedures developed for managing patient safety at Mindspot. We also conducted a retrospective observational study of the number of patients who self reported suicidal ideas and plans for patients who completed assessments, and the circumstances and characteristics of patients who were urgently referred to local services in a full year of operation of MindSpot.

The anonymised details of all patients who, as part of their assessment, gave written consent for the data they provided to be analysed by confirming agreement with the terms and conditions of use during the initial assessment, were extracted for the year from July 1, 2013 to June 30, 2014. MindSpot began operation in December, 2012, but the period of analysis was chosen because there were a number of changes to the assessment forms and to the procedure for recording emergency responses in the first six months of operation.

The clinic assessment and treatment data base was searched for the number of patients who reported suicidal ideas and suicidal plans, the scores on self report questionnaires for distress (K-10) and depression (PHQ-9), and the number of patients referred by MindSpot for urgent face to face care. Mindspot is limited to people aged over 18.

A summary of the contact with the patient and local services and a copy of the referral letters were kept in a crisis management file for all patients who were referred for urgent care. The referral letters include the patients’ demographic details and a summary of their reported symptoms, including their scores on the K-10 and the PHQ-9 for comparison with the overall clinic sample. Data was extracted from those documents.

Statistical analysis was limited to reporting frequencies and mean scores on the K-10 and PHQ-9, using Excel.

The paper was prepared in accordance with the NH&MRC Guidelines for reporting routine clinical care and the approval granted by the research and ethics committee of Macquarie University for a prospective uncontrolled observation study of the outcome of MindSpot patients.

## Results

### Assessing risk of suicide during online assessment

The initial contact for most patients accessing MindSpot is through an online assessment, using a structured questionnaire to identify presenting problems, and clinically validated questionnaires to elicit the overall level of psychological distress (K10) [13], symptoms of depression (PHQ9) [14], anxiety (GAD-7) [15], and where indicated symptoms of social phobia (SPIN) [16], panic disorder (PDSSR) [17] obsessive compulsive disorder (YBOCS) [18] and post-traumatic stress disorder (PCL) [19]. Every person who completes an assessment is then contacted with the results of the self-assessment, and offered admission to one of four MindSpot courses or supported to learn about and access local mental health services. People who complete assessments can elect to be contacted by email only, and can also choose not to disclose their identity.

At the end of the assessment questionnaires, patients are asked to answer questions designed to identify the presence of suicidal thoughts, plans and intent. These questions include whether they have thoughts that life is not worth living, and whether they plan or intend to end their lives. Those responding “yes” to one or both of those questions receive an immediate on-screen alert which encourages personal safety and provides contact details for crisis and emergency services. These people are then contacted as soon as practicable by telephone by a MindSpot therapist who verbally administers a structured risk assessment which asks in more detail about suicidal thoughts and behaviour, risk factors, previous suicide attempts, access to lethal means of suicide and the imminence of any plan. These questions are based on those used by the New South Wales Health framework for suicide risk assessment and management (http://www.health.nsw.gov.au/mhdao/publications/Pages/suicide-risk.aspx) and are consistent with the standards set out in the National Quality Framework for Telephone Counselling and Internet Based Support Services (http://www.health.gov.au/internet/main/publishing.nsf/Content/mental-pubs-q-quatel). Patients who have elected to be contacted by email only still receive a telephone call, as the terms of use make it clear that telephone contact would be attempted in the event of concerns about safety. The questions are taken from an on-screen form seen by the therapists only, to ensure therapists adhere to the risk assessment protocol and ask the key questions. The risk assessment procedure includes the completion of a crisis summary report, which summarises the risk and protective factors, reports on referrals or other actions taken in response to the assessed risk, and records the results of those actions.

Those patients who report immediate risk, in that they are unable to guarantee their safety for at least 24 hours, or who the therapist has reason to believe is at imminent risk, are referred for urgent assessment by a mental health crisis team or are referred immediately to emergency services (See Additional file 1: Figure S1). After the risk assessment all patients who do not require immediate support are then asked to develop a safety plan based on existing or potential support networks. This plan identifies at least three people or services the person agrees to contact if symptoms return or worsen. All patients are provided with the contact details for Lifeline and a 24 hour suicide call back service as part of the feedback. Details of the Safety Plan are documented in the Assessment Report, which is sent to the patient.

### Assessing risk in patients who are in online treatment

Similar risk assessment procedures are followed with patients engaged in one of the treatment courses offered at MindSpot. Triggers for administration of the structured risk assessment for patients in treatment include the patient reporting that they have developed suicidal intention or plan in the PHQ-9 questionnaire, which is automatically administered when the patient logs on each week, or if the total score on the PHQ9 rises more than five points, or above a pre-determined cut off score of 20, indicating a high level of depression [14]. The emergence of suicidal thoughts, or elevations in scores, trigger automated alerts that inform the therapist and senior MindSpot staff, and are followed by attempts to contact the patient.

### Clinical governance, supervision and training

All MindSpot therapists, most of whom are registered psychologists, receive competency-based training in administration of risk assessments, as well as training in how to engage with crisis or emergency services when referring patients for immediate support. The shift supervisor and other senior clinical staff, including the clinic psychiatrists, are alerted by therapists whenever a risk assessment is conducted and support the therapist during the assessment by providing immediate clinical supervision and support in identifying and contacting relevant mental health crisis and emergency services near the patient. Each crisis summary report is checked and confirmed by the senior therapist on duty (Shift Supervisor).

### Participants

In the year from July 1, 2013 to June 30, 2014, 9061 unique patients completed assessments at MindSpot and gave permission for analysis of their data. Of these, 2599 patients enrolled in a MindSpot treatment course. The mean K-10 scores for all those who completed an assessment was 32.3, and the mean PHQ-9 score was 15.6, which are scores associated with a high level of psychological distress [13] and moderate to severe depression [14]. Those who completed assessments, including a safety plan where indicated, were invited to join one of four treatment courses; the Wellbeing Course, a transdiagnostic course for anxiety and depression, the Wellbeing Plus course, a transdiagnostic treatment course for older adults, and courses for post traumatic stress disorder and obsessive compulsive disorder, or were supported to seek face to face mental health care. There was no cut off score for participation in a course, and the choice of course depended on the patient’s age and the pattern of symptoms confirmed in follow up communication. The results of the first year of treatment are reported in detail elsewhere [20].

At the end of the assessment process the patients were asked a series of questions to establish the presence of past suicidal behaviour, thoughts and plans, including whether they had made a suicide attempt in the last five years, whether they had experienced suicidal thoughts in the last year, and whether they had a current plan to commit suicide. A total of 2366 patients (26.1 %) acknowledged having thought about suicide, and 213 (2.4 %) also reported either intending to commit suicide or having a plan to do so. All of the patients who reported having suicidal intent or a plan were contacted by telephone and most were able to complete a safety plan to the satisfaction of MindSpot staff, which in each case was confirmed in supervision with a clinic manager. However, a small number of those patients were deemed to remain at elevated risk of suicide, either because of the nature of their condition as assessed by the clinician, or because of the responses they gave when questioned in more detail, during which they were unable to provide a satisfactory safety plan or assurances to the clinic staff. Those patients were immediately referred to either local mental health services, or to emergency services.

There were 51 patients referred for urgent care because of self reported suicidal plans and being unable to provide satisfactory guarantees of their safety, including 31 females and 20 males, ranging in age from mid teens to mid seventies. It should be noted that while MindSpot does not aim to provide services to people aged under 18, it does support younger people to access appropriate services, including support in the event of a crisis. All but two of the patients provided their residential addresses, which were from all states of Australia and one from the Northern Territory. Most of patients requiring urgent referral were from capital cities and major regional centres, but 19 (37 %) were in rural or remote locations. The mean score of the urgent referrals on the K-10 was 39.7, which is in the very high range [13], and the mean score on the PHQ9 was 22.1, indicating severe depression [14].

Most of the crisis referrals were made at the time of assessment, with only three patients referred for urgent care as a result of crises emerging while participating in a treatment course. The most common reason for crisis referral was the clinician’s concern that the patient was at risk of suicide. One patient was believed to have an untreated psychotic illness. All but four of the patients assured the telephone assessor that they did not intend to commit suicide immediately and would accept urgent referral. Of those four patients, one reported that she had taken an overdose of medication, and an ambulance was called and attended her home while the therapist was in telephone contact, and three patients were referred to police in Queensland, Victoria and Tasmania respectively to check on their safety.

Of the two cases in which the patient refused to supply their residential address, one was a teenage boy in a capital city, who agreed to contact a specialist youth mental health service, and the name and mobile telephone number of the patient who did not provide an assurance that he did not intend to commit suicide were provided as an urgent referral to the Queensland Police.

Of the remaining patients, all accepted referral to local health services. Two patients, one in a remote location, were referred to general practitioners and the remaining patients were referred to the community mental health teams serving their areas. In each case we were able to identify the responsible service, confirm the receipt of the written referral and handover of care, and in most cases were able to confirm that the local service had made contact with the patient. Where possible, the patients’ general practitioners were also sent copies of the urgent referral letters. The average amount of therapist time required to make an acute referral was calculated to be approximately three hours, including telephone calls made to the patient, discussions with supervisors and time spent locating and contacting local health services. (Table 1).

**Table 1:** Summary of MindSpot patients

Total number of patients who completed an online assessment	9061
Number who reported suicidal ideation	2366 (26.1 %)
Number reporting suicidal plans	213 (2.4 %)
Number reporting suicidal plans and unable to complete a satisfactory safety plan (urgent referrals)	51 (0.6 %)

## Discussion

The results of a full year of operation showed that the people who sought assessment at MindSpot had high levels of psychological distress and symptoms of depression, with mean scores that were well into the clinically significant range [20]. Suicidal thoughts are a common symptom of depression, and about a quarter of the patients assessed by the MindSpot report suicidal thoughts. The rate of suicidal ideation is far higher than the rate reported in the 2007 Australian National Mental Health and Wellbeing Survey, in which 3 % reported suicidal ideas in the previous year [21]. It is possible that people are more willing to disclose the presence of suicidal thoughts during an online questionnaire than in a face to face interview [11], although it is also possible that those people know not to disclose suicidal thoughts in other settings. However, the high levels of symptoms in the completed assessments show that MindSpot assessed and treated people with significant illness.

Although expressed suicidal thoughts have not been shown to be a predictor of subsequent suicide in mood disorder [7] the disclosure of thoughts of suicide in people who have also disclosed high levels of symptoms of depression required further investigation and action. Most of the patients who reported suicidal thoughts and plans were able to be contacted by telephone, acknowledged that the thoughts were part of a depressive illness and were cooperative with the therapists in preparing a safety plan in preparation for commencing treatment for depression. Those patients were able to describe the supports they would make use of in the event of the emergence of strong suicidal thoughts or plans, and were cooperative with the therapists in devising Safety Plans to the satisfaction of the therapists and the shift supervisors.

A small proportion was deemed to require acute referral for face to face care. The results of this audit of the outcome of acute referrals made by MindSpot showed that there were only a small number of patients in that category, around 0.6 % of patients who completed assessment, that most were willing to accept referral from our service, and local services were readily available all over Australia. This review demonstrated that our initial concerns about having to deal with patients threatening immediate suicide but who would then refuse to cooperate with referral or provide contact details were largely unfounded. The concern that local services would not accept referrals from MindSpot, and the therapists would spend a large proportion of their time dealing with crises also proved to be unfounded. Despite a relatively high proportion of patients with high levels of psychological distress and symptoms of depression, actively suicidal patients were a small but important proportion of all the patients who contacted MindSpot seeking treatment for anxiety and depression. The cooperative attitude of most patients may reflect the care seeking nature of patients who have gone to the trouble of contacting MindSpot and completing an assessment.

Many of the people who completed an assessment did not want to enrol in a treatment course, and were provided with the results of the assessment, information about the types of treatment that was available and support to access to other forms of care. In that sense, one of the functions of MindSpot was to act as a national triage service, to allow patients to identify their own symptoms, and to assist patients who did not want to join one of the treatment courses to find other forms of care. The majority of patients with clinically significant symptoms who completed one of the courses moved from clinical to subclinical levels during the course, and most maintained those gains at three months [20]. The patients who did not improve were advised to seek review by their general practitioner or were supported to access face-to-face care. Hence, in addition to treating anxiety and depression, an important function of MindSpot was to encourage people to seek the treatment commensurate with their reported level of symptoms, either at the time of assessment, or during or after completing treatment.

Limitations to the study included that about 5 % of those who completed assessments did not give permission for their de-identified data to be used for research and evaluation, and we have not examined whether those patients were typical of the overall sample in other respects. We were also unable to confirm that the reports in the Clinic’s crisis management folder was a complete sample of the acute referrals. A further limitation is that we were unable to establish in every case that the patient had been contacted, or the outcome of the crisis referral. Therapists were made aware of several patients who had acute admissions to hospital as a result of referrals to local services. However, we were not aware of any suicides or other serious adverse events among people who had sought assessment at MindSpot. In most cases patients were willing to accept acute referral. However, in some cases the acute referral was made for patients who could not be contacted, and their views of the referral could not be established.

## Conclusions

Around 0.6 % of the people seeking assessment or treatment by MindSpot were referred to local mental health services for urgent face to face care. The procedures for identifying and managing those patients were satisfactory, and in every case, either emergency services or local mental health services were able to take over the patient’s care. A review of acute patients assessed by an online treatment service shows that the uncertainty associated with taking responsibility for the remote treatment of patients who disclose active suicidal plans is not a major impediment to providing direct access online treatment for severe forms of anxiety and depression.

## Additional file

Additional file 1:
**Flow chart for responding to potentially suicidal patients.** (DOCX 84 kb)
