# Supplementary material for: Procedures for risk management and a review of crisis referrals from the MindSpot Clinic, a national service for the remote assessment and treatment of anxiety and depression
Source: BMC Psychiatry. 2015 Dec 1;15:304. doi: 10.1186/s12888-015-0676-6 (PMC4666146; doi:10.1186/s12888-015-0676-6)
Supplement: Additional file 1: — Flow chart for responding to potentially suicidal patients. (DOCX 84 kb) [file 12888_2015_676_MOESM1_ESM.docx]

**HIGH RISK CALL FLOW CHART**

**DETERMINE LEVEL OF RISK**

**Can you keep yourself alive for the next 24 hours?**

Question to determine **IMMINENCE**:

1. Current access to means
2. Unable to guarantee safety for the next 24 hours

**NO - IMMINENT RISK**

YES - HIGH RISK

Discuss Crisis Support Plan with Patient

Patient agree to comply with Crisis Support Plan during discussion

Need to Ensure:

1. Patient is co-operative and well supported
2. Crisis Support plan provides strategies to keep patient safe in event of crisis (ex. GP, 000, Lifeline)
3. Contact arranged in a week for follow-up unless commencing treatment within the next week

Patient does **NOT** agree to comply with crisis support plan during discussion

Discussion becomes clear that Patient is at imminent risk

**IMMINENT RISK**

**Refer to IMMINENT RISK Flow Chart**

**and**

**CHECK THAT YOU KNOW PATIENT’S LOCATION**

**IMMINENCE RISK CALL**

Are you willing to be in contact with a crisis service within the next hour? Y/N

Y: Patient agrees to contact or provides consent to have us contact a crisis service

N: Patient does **NOT** agree or consent to us contacting a crisis service

Duty of Care reinstated

- Not a crisis service
- “We need to ensure you stay safe”

ON PHONE WITH PATIENT:

Arrange for warm follow-up with the patient 1 hour later (try get home and mobile number)

Inform that if unable to reach, we will call the local mental health team

**IMMINENT THREAT**

**(Within 1 Hour)**

**“I’m going to do it”**

Contact Local Mental Health Team, **get a contact name and contact details**, then complete urgent referral form

**Keep patient on the phone, and be warm, supportive and keep them engaged**

Call Patient 1 hour later and

**NOT AVAILABLE**

- Call once. If no

answer, call again 10 minutes later

Call Patient 1 hour later and **AVAILABLE**

1. Determine if contacted by crisis service
2. Triage by re-assessing for imminent risk
3. Provide crisis contact numbers again
4. Inform patient about contact with Mental Health Team and next steps

**Call Police on 000**

**or**

**Local Mental Health Team**

If unreachable;

- Contact patient’s local Mental Health Team
- Follow-up email to patient

Follow-up email to patient with support plan including crisis plan details
